# Multimodal Branched Transport Infers Anatomically Aligned Brain Reaction Maps

**DOI:** 10.1007/s12021-026-09791-4

**Published:** 2026-06-10

**Authors:** Cristian Mendico

**Affiliations:** Institut de Mathématique de Bourgogne, UMR 5584 CNRS, Université Bourgogne Europe, Dijon, France

**Keywords:** Branched optimal transport, Brain networks, Multimodal neuroimaging, Connectomics, Stochastic dynamics, Structure–function coupling

## Abstract

How external stimulation is transformed into distributed reaction patterns remains unresolved at the level of propagation architecture. Existing large-scale control models quantify transition costs on prescribed networks but do not infer the routing map itself from source and target activity. Here we combine task-related blood-oxygen-level-dependent responses, source-reconstructed electrophysiology and tractography-derived anisotropy to estimate stimulation and reaction measures, define an anatomical transport cost, and infer a branched propagation architecture by variational optimisation. Unlike standard transport formulations, branched transport favours aggregation of signal into shared neural highways before redistribution. We further equip the inferred map with a stochastic graph-induced dynamical model to the inferred map and quantify the trade-off between geometric efficiency and dynamical controllability. We show that multimodal data generate anatomically aligned brain reaction maps, that anisotropic costs qualitatively reshape routing backbones relative to isotropic baselines, and that hybrid geometric–dynamical optimisation reveals non-trivial rank reversals across branching regimes.

**Supplementary Information:**

The online version contains supplementary material available at 10.1007/s12021-026-09791-4.

## Introduction

A central problem in systems neuroscience is to determine how an external stimulation is propagated through the brain so as to produce a reaction. Current deterministic and stochastic control approaches quantify the cost of driving brain states on a prescribed structural substrate, but they do not treat the routing architecture itself as an unknown (Gu et al., 2015; Kamiya et al., 2023; Kawakita et al., 2022). In the companion theoretical paper, we proposed a different variational viewpoint: the unknown is a graph or current connecting a stimulation source measure to a reaction target measure, selected as the minimiser of an anisotropic branched transport problem (Mendico, 2026). In that framework, the support of the minimiser defines a *brain reaction map*, namely an inferred stimulus-to-reaction routing architecture rather than a controlled trajectory on a fixed network (Mendico, 2026).

The present article develops the data-driven computational counterpart of that theory. Instead of assuming abstract source and target measures, we estimate them from multimodal neuroimaging signals. Instead of postulating a generic transport metric, we derive a geometry-aware cost from tractography-informed anisotropy (Hagmann et al., 2007; Yeh et al., 2021; Zhang et al., 2022). Instead of stopping at the geometric minimiser, we use the inferred graph as the substrate of a stochastic whole-brain dynamics and evaluate the trade-off between anatomical transport efficiency and dynamical control cost. This places the work at the intersection of multimodal neuroimaging, connectomics and large-scale brain modelling (Breakspear, 2017; Deco & Kringelbach, 2020; Gilson et al., 2020; Pathak et al., 2022; Patow et al., 2024).

A useful way to situate the present framework within computational neuroscience is to compare it with standard approaches to directed or effective connectivity inference. Granger-causal methods and dynamic causal modelling aim to infer directed interactions among brain regions from observed time series, typically by estimating lagged statistical dependencies or by fitting state-space models with hidden neuronal variables and modality-specific observation equations (Friston et al., 2013; Novelli et al., 2024). These approaches have been highly influential for the study of functional integration, but they address a different inverse problem from the one considered here: they infer coupling parameters on a preselected set of nodes and candidate interactions, whereas our variational problem infers a transport backbone connecting source and target measures under an anatomically anisotropic cost. In this sense, the object inferred here is not primarily an edgewise effective-connectivity matrix, but a stimulus-to-reaction routing architecture selected by conservation, anisotropy and branching economy.

The present work is also related to recent neuroinformatics and causal-discovery approaches that reconstruct brain connectivity models from fMRI or multimodal recordings (Saetia et al., 2021). Such methods are valuable for exploratory graph inference and for disentangling direct from indirect statistical pathways, but they remain conceptually distinct from the BOT construction proposed here. Our model does not seek a generic causal graph from time series alone. Instead, it starts from a source–target representation of the stimulus-to-reaction transformation, incorporates tractography-informed directional priors, and selects a graph by minimizing a concave transport functional that explicitly favours shared relay corridors. This is also the key point of departure from network-control and whole-brain dynamical frameworks (Gilson et al., 2020; Gu et al., 2015; Kamiya et al., 2023; Kawakita et al., 2022; Patow et al., 2024): in those settings one studies trajectories, controls, or effective couplings on a prescribed substrate, whereas here the substrate itself is part of the inference problem. We therefore view the proposed framework as complementary to existing effective-connectivity, causal-discovery and whole-brain modelling approaches: it addresses the upstream question of which anatomically plausible routing architecture best links stimulation to distributed reaction.

This leads to three concrete questions. First, can task-related blood-oxygen-level-dependent responses and source-reconstructed EEG/MEG be fused into coherent source and target measures for a transport problem? Second, does tractography-derived anisotropy substantially alter the inferred routing architecture relative to isotropic baselines? Third, once a graph is inferred, is the geometrically optimal map also dynamically plausible as a substrate for stochastic state transitions?

To answer these questions, we implement the full multimodal pipeline already sketched in the theoretical work (Mendico, 2026): (i) estimation of the source and the target measures from fMRI and EEG/MEG, (ii) construction of an anisotropic transport cost from diffusion-informed tensors, (iii) branched transport optimisation on a candidate graph, and (iv) a hybrid stochastic extension built on the inferred routing architecture. The resulting article is intentionally complementary to the theoretical companion paper: the theory establishes the variational mechanism (Mendico, 2026), whereas the present work demonstrates that the mechanism yields biologically interpretable, anatomically constrained and dynamically non-trivial reaction maps in a multimodal setting.

## Theoretical Construction Underlying the Brain Reaction Map

The present computational study is based on the variational construction introduced in the companion theoretical paper (Mendico, 2026). We briefly recall here the main objects and the modelling principle, in order to make the data-driven results below self-contained.

Let $\Omega$ denote the anatomical or mesoscale neural domain under consideration. The external stimulation is represented by a nonnegative source measure

$$ \mu ^+_\textrm{stim}\in \mathcal {M}_+(\Omega ), $$

whereas the reaction-producing neural configuration is represented by a nonnegative target measure

$$ \mu ^-_\textrm{react}\in \mathcal {M}_+(\Omega ). $$

The two measures are assumed to be balanced,

$$ \mu ^+_\textrm{stim}(\Omega )=\mu ^-_\textrm{react}(\Omega ), $$

so that the problem is formulated as a conservative transport problem from stimulation to reaction. In the data-driven setting of the present paper, these measures are not prescribed abstractly: they are estimated from task-related blood-oxygen-level-dependent responses and source-reconstructed electrophysiological activity.

The central modelling step is to treat the propagation architecture itself as the unknown. In the continuous formulation, one seeks a rectifiable transport current

$$ v=\tau \,\theta \,\mathcal {H}^1\!\llcorner M, $$

that is, an oriented one-dimensional transport structure describing where the signal propagates, in which direction, and with what intensity. Here $M\subset \Omega$ is a countably $1$-rectifiable set, $\tau$ is an orientation field, $\theta \ge 0$ is the transported flux multiplicity and $H^1$ is the one-Hausdorff measure. The current is constrained by the balance law

$$ \textrm{div}\,v=\mu ^+_\textrm{stim}-\mu ^-_\textrm{react}. $$

Thus, the current connects the stimulation distribution to the reaction distribution, and its support represents the effective propagation backbone.

The anatomical geometry enters through a positive anisotropic cost density

$$ c:\Omega \times \mathbb {S}^{d-1}\longrightarrow (0,\infty ), $$

which assigns a transport cost to moving at position $x$ in direction $\tau$. This density may encode local tissue geometry, tractography-derived anisotropy, preferred fibre orientation, structural connectivity, or other anatomical priors. For a fixed branching exponent $0<\alpha <1$, the anisotropic branched transport energy is

$$ \mathcal {M}_{\alpha ,c}(v) = \int _M c(x,\tau (x))\,\theta (x)^\alpha \,d\mathcal {H}^1(x). $$

The concavity of $m\mapsto m^\alpha$ is the key mechanism: transporting two signals together along a common segment is cheaper than transporting them separately. Consequently, minimizers are expected to form ramified, tree-like structures with shared corridors and branching points.

The continuous variational problem is therefore

$$ \min \left\{ \mathcal {M}_{\alpha ,c}(v): v\in \mathcal {M}(\Omega ;\mathbb {R}^d),\ \textrm{div}\,v=\mu ^+_\textrm{stim}-\mu ^-_\textrm{react} \right\} . $$

Under the compactness and positivity assumptions stated in the companion paper, this problem admits minimizers. The support of a minimizer $v^*$,

$$ M^*=\textrm{supp}(v^*), $$

is called the brain reaction map. It is interpreted as the preferred stimulus-to-reaction routing architecture selected jointly by the source–target configuration and the anatomical cost.

For the numerical implementation used in this article, we work with the corresponding discrete graph formulation. A finite candidate graph is constructed on the set of regions of interest. If $w_e\ge 0$ denotes the flux carried by a directed edge $e$, and if $A$ is the incidence matrix of the directed graph, the Kirchhoff constraint reads

$$ Aw=b, \qquad b=\mu ^+_\textrm{stim}-\mu ^-_\textrm{react}. $$

For each edge $e$, the anatomical-geometric cost is

$$ \beta _e = \int _0^{\ell _e} c\!\left( \gamma _e(s), \frac{\dot{\gamma }_e(s)}{\Vert \dot{\gamma }_e(s)\Vert } \right) \,ds, $$

where $\gamma _e$ is the embedded edge path. The discrete branched transport problem is then

$$ \min _{w\ge 0} \sum _{e\in E}\beta _e w_e^\alpha \quad \text {subject to} \quad Aw=b. $$

The optimal weighted support defines the discrete brain reaction map. Edges with large flux identify principal propagation routes, while vertices where several high-flux edges merge or split identify candidate relay or redistribution regions.

This construction differs from standard network-control approaches in an essential way. In classical deterministic or stochastic control models, the structural substrate is fixed in advance and one optimizes a trajectory or control signal on that substrate. Here, by contrast, the substrate itself is inferred from the stimulation and reaction measures. The optimization variable is not primarily a controlled trajectory, but a graph or current representing the routing architecture.

Finally, the companion theory also motivates the hybrid stochastic extension used below. Once a graph $(G,w)$ has been inferred, it induces a graph-dependent stochastic dynamics of the form

$$ dX_t = A_{G,w}X_t\,dt + B_\textrm{stim}a_t\,dt + u_t\,dt + C_{G,w}\,dW_t, $$

where $A_{G,w}$ contains a graph-Laplacian propagation term and $C_{G,w}$ encodes graph-dependent noise. The dynamic cost

$$ J_{\text {dyn}}(G,w) = \inf _u {\text {KL}}\!\left( P_{G,w,u}\,||\,P_{G,w,0} \right) $$

measures the minimum path-space control effort required to steer the graph-induced stochastic process between prescribed endpoint distributions. This leads to the hybrid functional

$$ F_\lambda (G,w) = E_\alpha (G,w)+\lambda J_{\text {dyn}}(G,w), $$

which balances geometric transport efficiency with dynamical plausibility.

The purpose of the present paper is to instantiate this construction from multimodal data. We estimate $\mu ^+_\textrm{stim}$ and $\mu ^-_\textrm{react}$ from fMRI and EEG/MEG features, derive anisotropic edge costs from diffusion-informed tensors, solve the discrete branched transport problem, and then evaluate the resulting reaction maps through the graph-induced stochastic dynamics.

## Results

### Task-evoked fMRI Defines Spatially Resolved Stimulation and Reaction Signatures

**Fig. 1:**
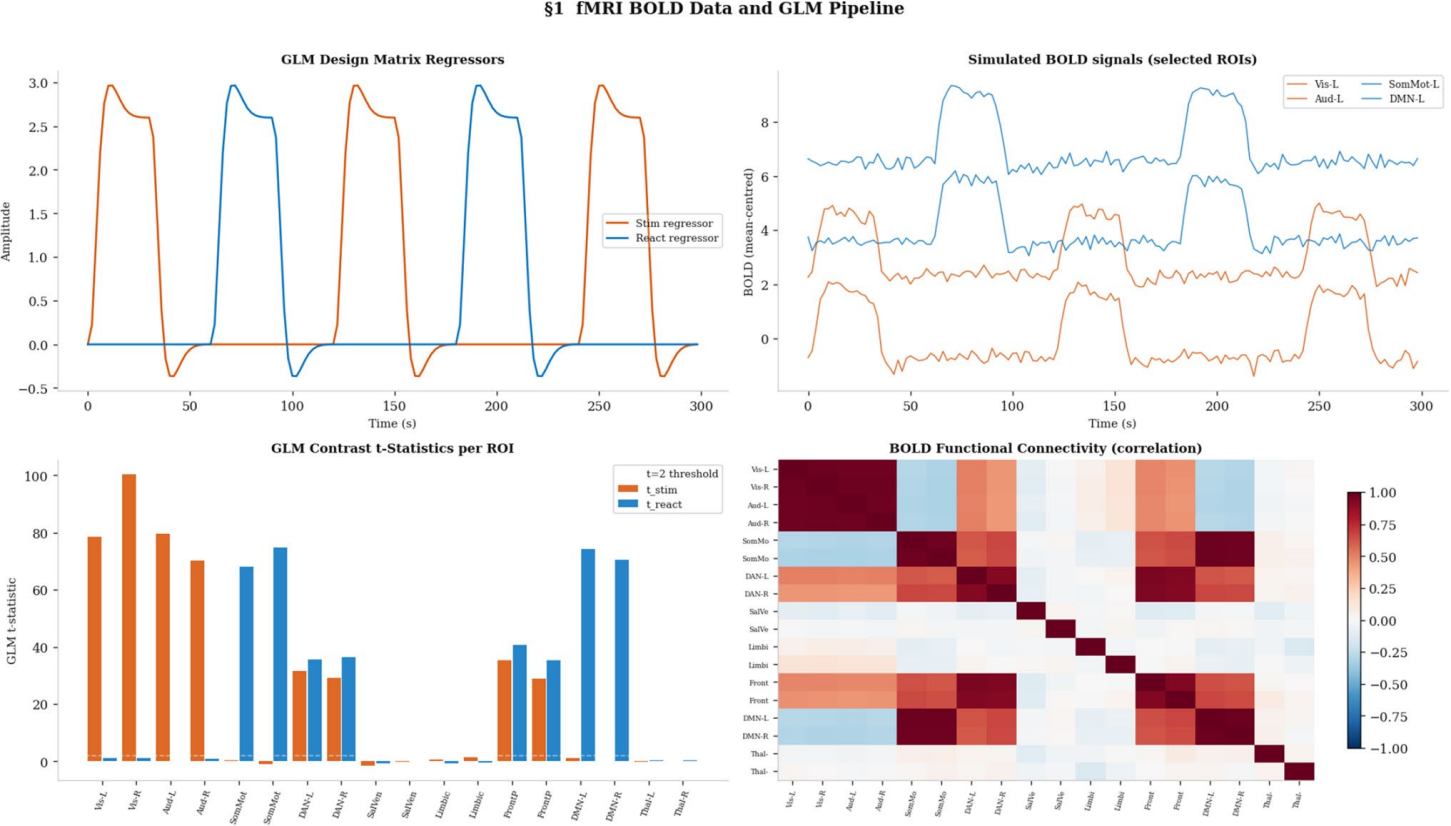
**Task-evoked fMRI BOLD pipeline.** Synthetic task-based blood-oxygen-level-dependent (BOLD) data were generated on an 18-region cortical support and analysed with a block-design general linear model. The left panel shows the stimulation and reaction regressors after convolution with the haemodynamic response function, illustrating the temporally separated experimental conditions used to estimate task-related activity. The upper-right panel displays representative regional BOLD time series, showing condition-dependent fluctuations across selected regions of interest. The lower-left panel reports region-wise contrast statistics for stimulation- and reaction-related effects, highlighting spatially selective activation patterns that distinguish early sensory-entry regions from later reaction-associated regions. The lower-right panel shows the resulting BOLD functional connectivity matrix, demonstrating that the simulated responses generate a non-trivial mesoscale correlation structure rather than independent regional activity. Together, these outputs define the fMRI-derived spatial component used to estimate the stimulation and reaction measures for the subsequent branched transport problem

We first simulated a task-based blood-oxygen-level-dependent experiment on a common cortical support with 18 regions of interest. As shown in Fig. 1, the block-design general linear model cleanly separates a stimulation regressor from a reaction regressor and generates region-wise contrast statistics with strong spatial selectivity. Early task epochs concentrate on visually and auditorily driven regions, whereas later task epochs emphasise sensorimotor and default-mode-related regions. The resulting blood-oxygen-level-dependent correlation structure is non-trivial and already exhibits mesoscale organisation rather than independent regional responses.

These outputs are important for two reasons. First, they show that the source and target measures can be grounded in standard task-based fMRI statistics rather than hand-crafted weights (Esteban et al., 2020; Friston, 2009). Second, they provide a spatially localised but temporally coarse characterisation of the stimulus-to-reaction transformation, which is precisely the role that fMRI should play in the multimodal pipeline.

### Source-Reconstructed EEG/MEG Resolves the Temporal Separation Between Stimulus and Reaction

**Fig. 2:**
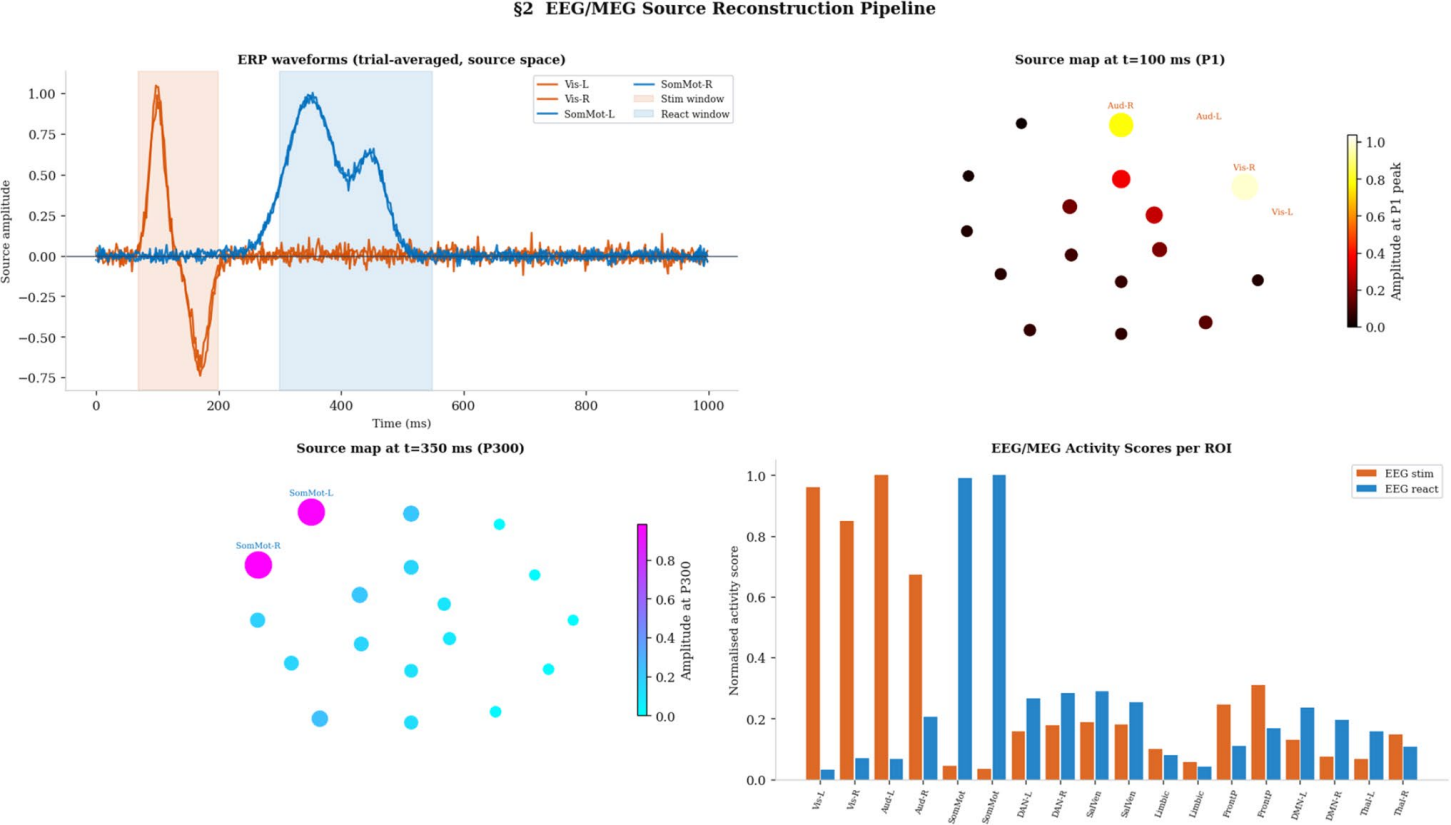
**Source-reconstructed EEG/MEG pipeline.** Synthetic electrophysiological recordings were generated using a lead-field forward model and reconstructed in source space with a minimum-norm inverse procedure. The upper-left panel shows trial-averaged source-space event-related potentials, with an early stimulus-locked component and a later reaction-related component. The upper-right panel displays the reconstructed source activity around 100 ms, corresponding to the early stimulation window and concentrating primarily in sensory-entry regions. The lower-left panel displays the reconstructed source activity around 350 ms, corresponding to the later reaction window and shifting toward motor and higher-order response regions. The lower-right panel summarises the resulting region-wise EEG/MEG activity scores for the stimulation and reaction windows. This figure provides the temporally resolved component of the source–target estimation pipeline, complementing the spatial localisation supplied by the fMRI analysis

We next constructed source-reconstructed electrophysiological data using a lead-field forward model and a minimum-norm inverse procedure. Figure 2 shows that this modality complements blood-oxygen-level-dependent data exactly in the way needed for the transport problem: early components concentrate in sensory entry regions, whereas later components dominate in reaction-related regions. The spatial source maps at approximately 100 ms and 350 ms reveal a clean temporal shift from stimulus-locked to reaction-locked activity.

This temporal separation is crucial. The source measure $\mu _{\textrm{stim}}^{+}$ should reflect entry of the external stimulation into the effective propagation architecture, whereas $\mu _{\textrm{react}}^{-}$ should reflect the later reaction-producing configuration. Blood-oxygen-level-dependent data alone localise these patterns but blur their timing; source-reconstructed EEG/MEG resolves them sharply. In this sense, Fig. 2 provides the temporal half of the inverse problem. The source-reconstruction viewpoint is standard in modern EEG/MEG analysis and is particularly useful when one aims to distinguish early stimulus-driven components from later reaction-related components (Knösche & Haueisen, 2022).

### Multimodal Fusion Yields Balanced Source and Target Probability Measures

We then combined the spatial specificity of blood-oxygen-level-dependent responses and the temporal precision of source-reconstructed electrophysiology through weighted geometric fusion of modality-specific activity scores. If $a_i^{\textrm{fMRI}}$ and $a_i^{\textrm{EEG}}$ denote normalised regional scores, the fused stimulation profile is

$$ a_i^{\textrm{stim}} = \bigl (a_i^{\textrm{fMRI,stim}}\bigr )^{w_f} \bigl (a_i^{\textrm{EEG,stim}}\bigr )^{1-w_f}, $$

with $w_f \in [0,1]$, and similarly for the reaction profile. After normalisation we obtain the balanced measures

$$ \mu _{\textrm{stim}}^{+}, \qquad \mu _{\textrm{react}}^{-}, \qquad b(v)=\mu _{\textrm{stim}}^{+}-\mu _{\textrm{react}}^{-}. $$

**Fig. 3:**
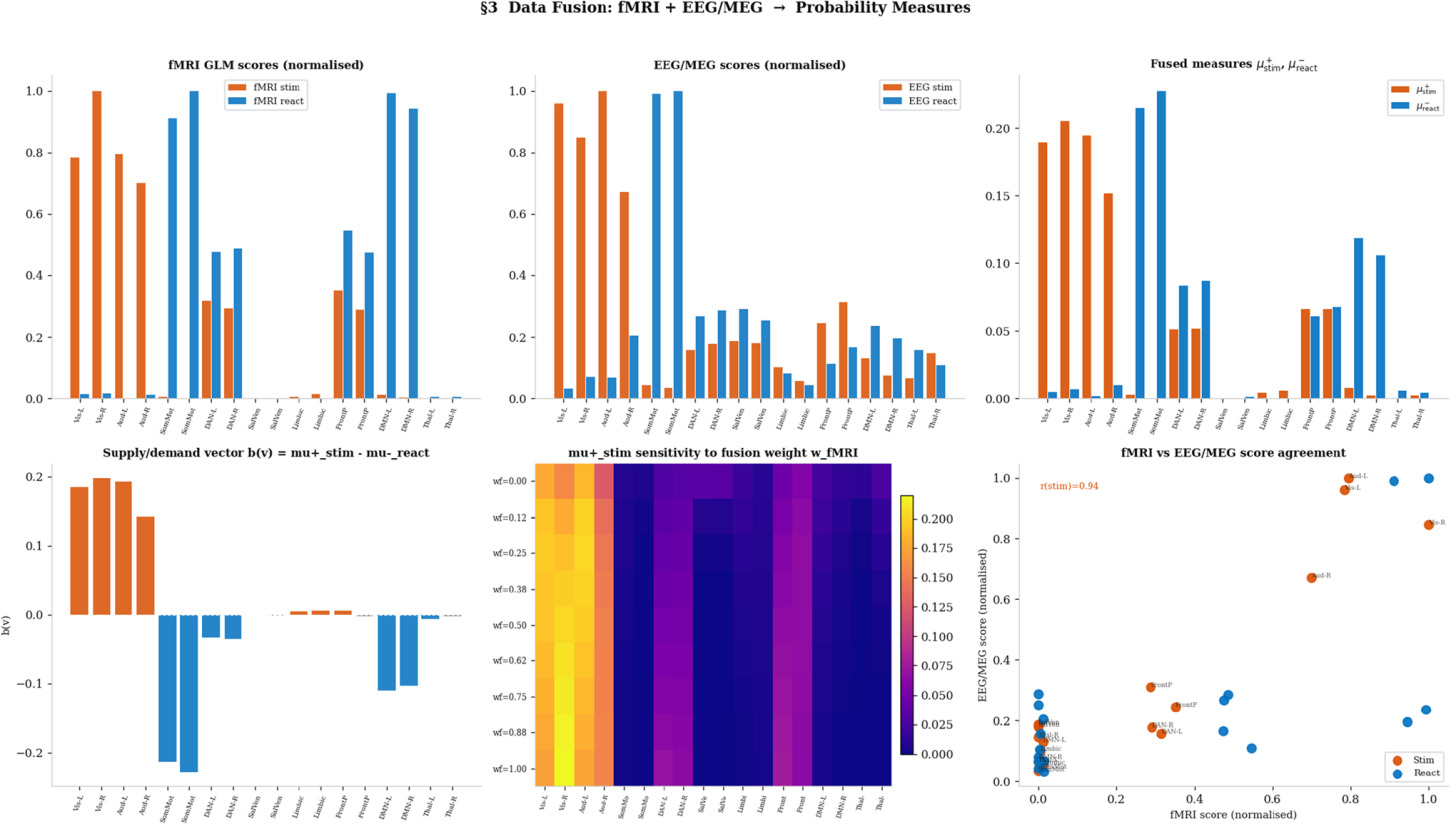
**Multimodal fusion and construction of balanced probability measures.** Spatially resolved fMRI scores and temporally resolved EEG/MEG scores were combined to define the source and target measures used in the branched optimal transport problem. The top row shows, from left to right, the normalised fMRI activity scores, the normalised EEG/MEG activity scores, and the fused stimulation and reaction profiles obtained by weighted geometric averaging of the two modalities. After normalisation, these fused profiles define balanced probability measures $\mu ^+_{\textrm{stim}}$ and $\mu ^-_{\textrm{react}}$ on the common regional support. The lower-left panel shows the supply–demand vector $b = \mu ^+_{\textrm{stim}}-\mu ^-_{\textrm{react}}$, which determines the Kirchhoff constraint in the discrete transport problem. The lower-middle panel reports the sensitivity of the stimulation measure to the multimodal fusion weight, indicating how regional mass is redistributed as the relative contribution of fMRI and EEG/MEG is varied. The lower-right panel shows the regional agreement between BOLD-derived and electrophysiology-derived scores. Overall, the figure demonstrates that multimodal fusion yields structured, balanced, and interpretable source and target measures rather than arbitrary input weights

Figure 3 shows the resulting modality-specific patterns and fused measures. The fused $\mu _{\textrm{stim}}^{+}$ remains concentrated on sensory entry regions, while the fused $\mu _{\textrm{react}}^{-}$ shifts toward motor and higher-order reaction regions. At the same time, intermediate relay regions acquire non-zero mass through cross-modal agreement rather than arbitrary assignment.

The lower row of Fig. 3 shows two additional points. First, the supply–demand vector *b*(*v*) is sharply structured, producing a transport problem that is neither trivial nor diffuse. Second, the sensitivity map with respect to the fusion parameter demonstrates that the dominant support is stable over a broad range of modality weightings, although quantitative mass redistribution persists in secondary regions. The transport problem is therefore not being driven by a fragile or modality-specific artefact. Additional analyses across the full $\alpha$-grid confirm that the transition from weakly aggregated to strongly aggregated routing is continuous over the stable parameter regime (Supplementary Figs. S1 and S2).

### Diffusion-Informed Anisotropy Induces a Directional Transport Geometry

To encode anatomical directionality, we assigned to each region of interest a synthetic diffusion tensor and used it to build a local anisotropic cost

$$ c(x,\tau )=\sqrt{\tau ^\top (D(x)+\varepsilon I)^{-1}\tau }. $$

For each candidate arc $e$, this induces a graph-level cost

$$ \beta _e = \int _0^{\ell _e} c\!\left( \gamma _e(s),\frac{\dot{\gamma }_e(s)}{\Vert \dot{\gamma }_e(s)\Vert }\right) \,ds, $$

approximated numerically by midpoint quadrature along the edge.

**Fig. 4:**
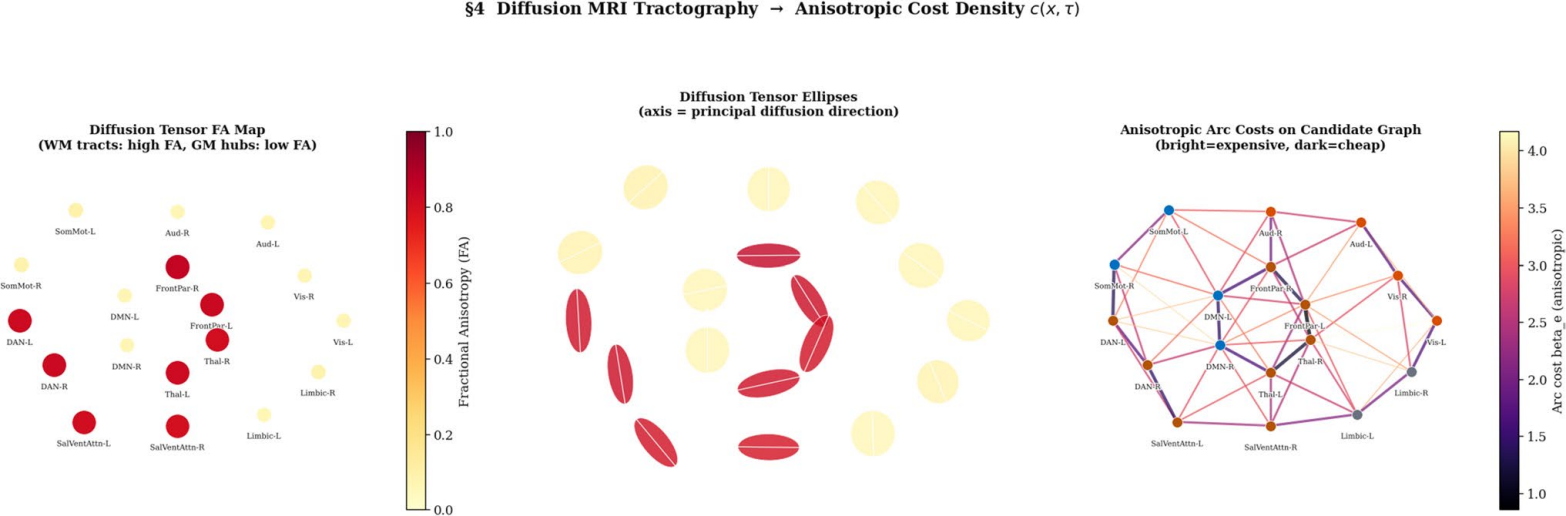
**Diffusion-informed anisotropic transport geometry.** A synthetic diffusion-informed tensor field was assigned to the cortical support in order to encode direction-dependent anatomical transport costs. The left panel shows the fractional-anisotropy values across regions of interest, with higher anisotropy assigned to relay-like regions intended to mimic white-matter-supported communication pathways. The middle panel displays the local diffusion tensor ellipses and their principal axes, illustrating the preferred directions of propagation imposed by the anatomical prior. The right panel shows the resulting anisotropic arc costs on the candidate graph. Because the cost of an edge depends not only on its Euclidean length but also on its alignment with the local tensor field, geometrically similar connections can acquire substantially different transport costs. This construction converts the routing problem from a purely distance-based optimisation into an anatomically informed inverse problem in which propagation is favoured along directions compatible with the diffusion-derived geometry

Figure 4 shows the resulting tractography-informed prior: white-matter-like relay regions have high fractional anisotropy, principal diffusion axes introduce directional preferences, and graph-level arc costs become heterogeneous even among geometrically similar connections. This construction is directly inspired by the use of diffusion-informed structure to constrain large-scale communication models in connectomics (Hagmann et al., 2007; Yeh et al., 2021; Zhang et al., 2022).

This step is decisive because it transforms the transport problem from a distance-based optimisation into a geometry-aware inverse problem. In the isotropic baseline, short edges are generically favoured. In the anisotropic model, some short edges become expensive if they cut across implausible directions, while longer edges can become favourable if they align with dominant tensor axes. Thus, the anatomical prior does not simply modulate the cost magnitude; it changes the directional logic of admissible propagation.

### Anisotropic Branched Transport Infers a Distinct Reaction-Map Backbone

We then solved the discrete branched transport problem

$$ \min _{w\ge 0}\; \sum _{e\in \mathcal {E}}\beta _e\,w_e^\alpha \qquad \text {subject to} \qquad Aw=b, $$

with $0<\alpha <1$, where the concavity of $w\mapsto w^\alpha$ promotes aggregation and branching (Mendico, 2026; Xia, 2003).

**Fig. 5:**
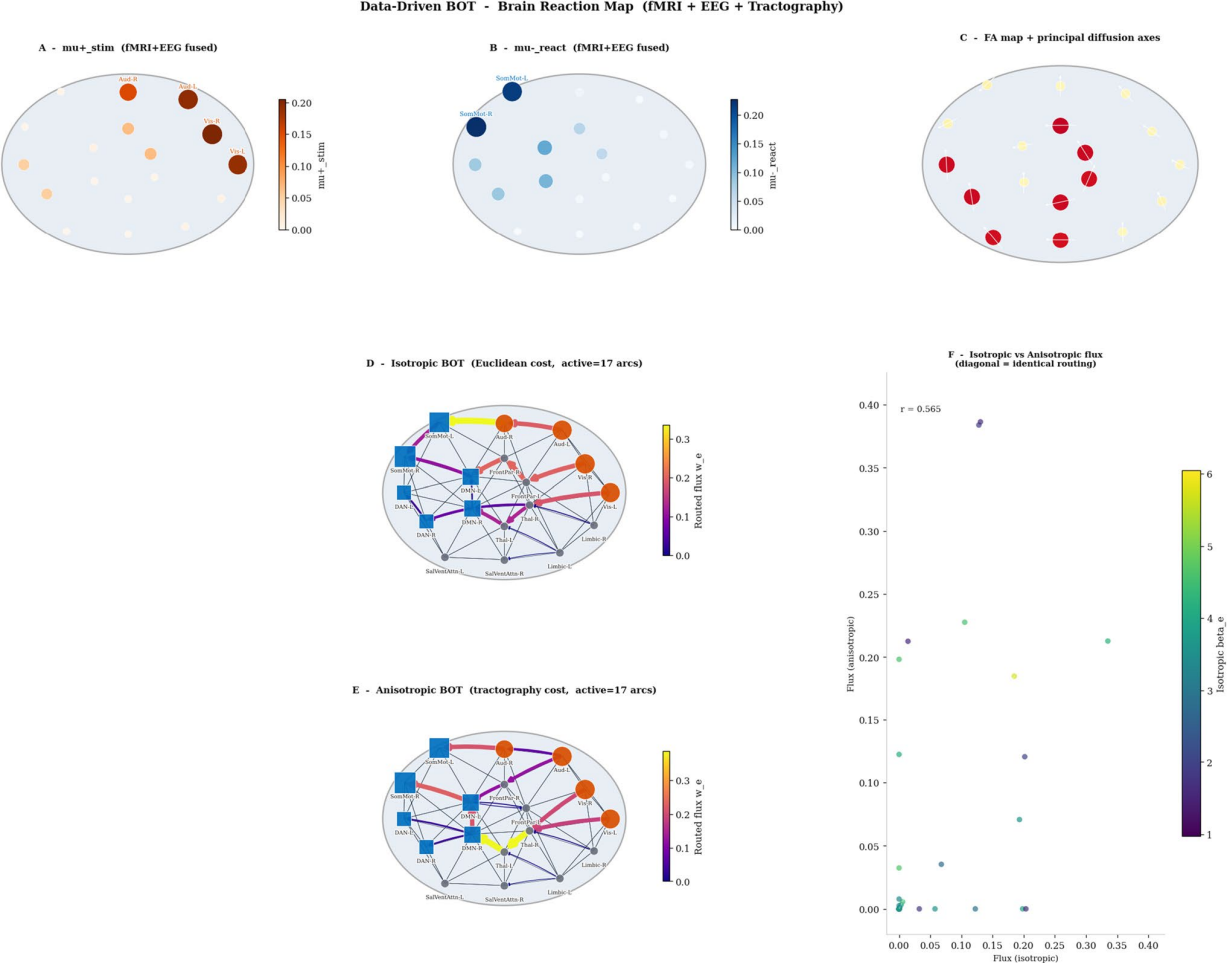
Data-driven branched optimal transport inference of the brain reaction map. The discrete branched optimal transport problem was solved on the candidate graph using the fused stimulation and reaction measures together with isotropic or anisotropic edge costs. Panels (**A**) and (**B**) show the fused source measure $\mu ^+_{\textrm{stim}}$ and target measure $\mu ^-_{\textrm{react}}$, respectively. Panel (**C**) shows the fractional-anisotropy map and principal diffusion axes used to define the anisotropic transport geometry. Panel (**D**) displays the isotropic branched transport solution, in which routing is driven primarily by geometric proximity. Panel (**E**) displays the anisotropic branched transport solution, in which the inferred propagation backbone is reorganised by the diffusion-informed directional prior. Panel (**F**) compares edgewise fluxes between the isotropic and anisotropic solutions, showing that anisotropy induces substantial redistribution of transported mass rather than a small perturbation of the isotropic map. The anisotropic model therefore selects a qualitatively distinct brain reaction map, with stronger alignment to anatomically plausible relay corridors and directional propagation structure

Figure 5 is the central result of the paper. The isotropic solution yields a branched architecture but remains relatively close to direct geometrical routing. The anisotropic solution, by contrast, reorganises the routing backbone around relay regions aligned with the tractography-derived geometry. Several branches that are only weakly expressed in the isotropic case become dominant under anisotropic costs, while some direct alternatives disappear entirely.

The flux comparison in Fig. 5f shows that the two solutions differ not only by small perturbations in weight but by substantial redistributions of mass across edges. In other words, anisotropy qualitatively reshapes the inferred reaction map. This is exactly the type of effect that fixed-substrate control models cannot reveal: once the substrate is prescribed, anatomical anisotropy can modulate dynamics on that substrate, but it cannot alter which routing architecture is selected as the canonical backbone in the first place. Comparison with simpler non-branched baselines further shows that the concave transport cost is specifically responsible for the emergence of shared relay corridors (Supplementary Fig. S3).

### Hybrid Stochastic Dynamics Reveals Geometric–Dynamical Trade-Offs and Rank Reversals

Finally, we used the anisotropic optimal graph as the substrate of the hybrid stochastic extension. On the inferred graph we defined a linear stochastic dynamics of the form

$$ dX_t = A_{G,w}X_t\,dt + B_{\textrm{stim}}a_t\,dt + u_t\,dt + C_{G,w}\,dW_t, $$

with

$$ A_{G,w}=-\kappa I-\beta _{\textrm{dyn}}L_{G,w}, \qquad C_{G,w}=\sigma _0 I+\sigma _1 D_{G,w}^{1/2}. $$

The associated hybrid functional is

$$ F_\lambda (G,w)=E_\alpha (G,w)+\lambda J_{\textrm{dyn}}(G,w). $$

**Fig. 6:**
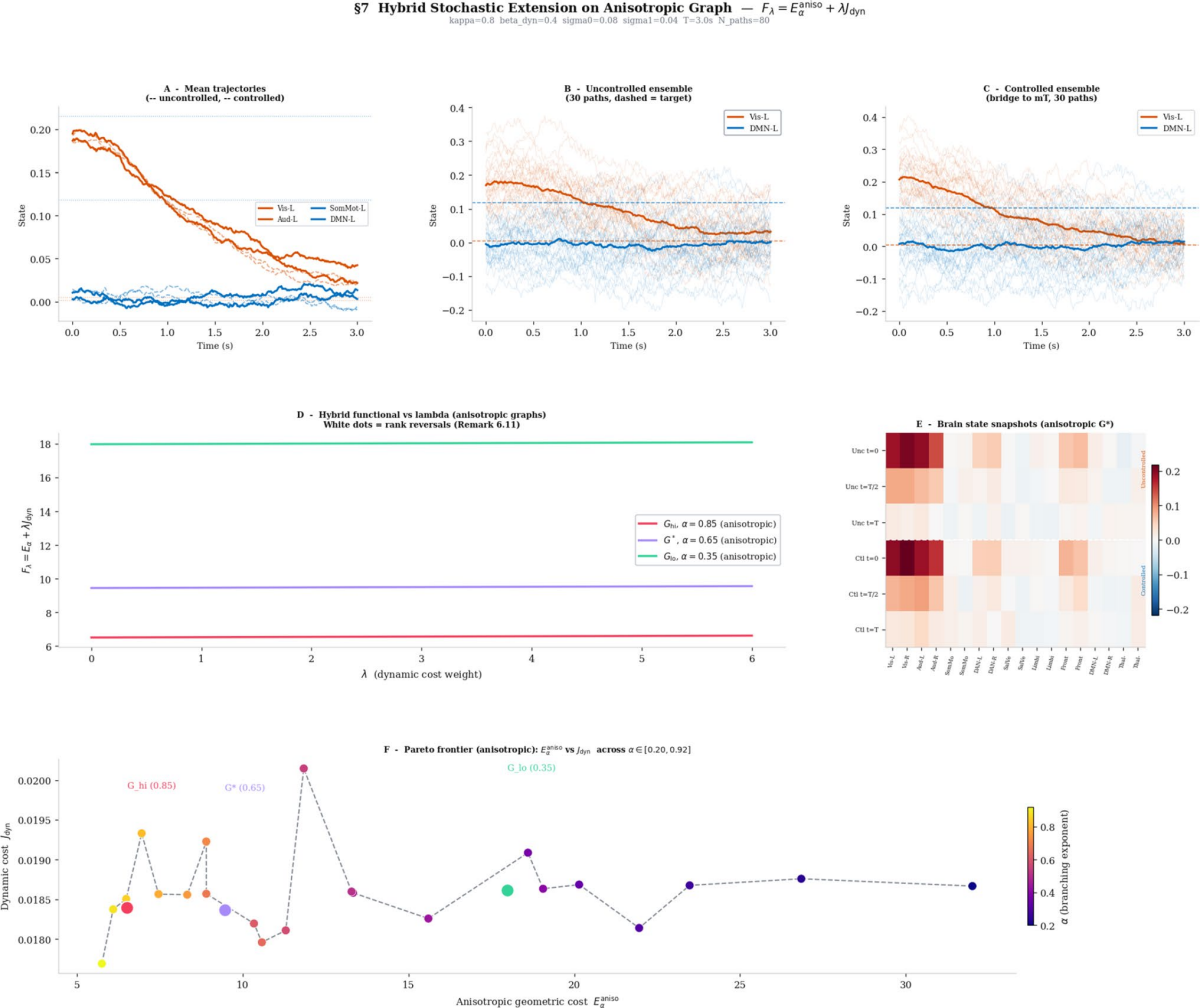
**Hybrid stochastic extension on the inferred anisotropic graph.** The anisotropic branched transport solution was used as the substrate for a graph-induced stochastic dynamical system in order to evaluate whether geometrically efficient reaction maps are also dynamically plausible. Panel (**a**) compares mean trajectories under uncontrolled and controlled dynamics, showing that feedback control steers the process closer to the prescribed reaction profile. Panels (**b**) and (**c**) show path ensembles for the uncontrolled and controlled stochastic processes, respectively, illustrating the reduction in dispersion and improved targeting under control. Panel (**d**) reports the hybrid functional $F_\lambda (G,w)=E_\alpha (G,w)+\lambda J_{\textrm{dyn}}(G,w)$ for competing graphs as the dynamical weight $\lambda$ is varied; crossings between curves indicate rank reversals between candidate reaction maps. Panel (**e**) shows representative brain-state snapshots along the stochastic evolution. Panel (**f**) displays the Pareto frontier in the plane of geometric transport cost and dynamic control cost across branching exponents $\alpha$. The figure shows that geometric efficiency and dynamical controllability are related but non-equivalent criteria, motivating the use of a coupled geometric–dynamical selection principle for brain reaction maps

Figure 6 shows three complementary results. First, controlled stochastic trajectories reach terminal states substantially closer to the prescribed reaction profile than uncontrolled trajectories, confirming that the inferred graph is dynamically usable as a state-transition substrate. Second, the hybrid functional reveals that graphs with lower geometric cost are not necessarily dynamically preferred. Third, the Pareto frontier across branching exponents $\alpha$ exhibits a non-trivial geometry, with local trade-offs between ramification and dynamic cost.

The geometric meaning of Fig. 6f is intuitive: each point corresponds to one candidate routing map, moving left reduces geometric transport cost, and moving down reduces dynamic control cost. The Pareto frontier is the set of non-dominated maps for which one criterion cannot be improved without worsening the other. Most importantly, Fig. 6d shows rank reversals across $\lambda$. As the weight of the dynamical term increases, the ordering of candidate graphs changes. A graph that is geometrically optimal can lose to a dynamically cheaper competitor, and vice versa. This is a strong argument against using purely geometric or purely dynamical criteria in isolation. The natural object is the coupled geometric–dynamical landscape. The graph-induced control viewpoint is consistent with recent work on stochastic steering and Schrödinger bridge formulations for brain state transitions (Kamiya et al., 2023; Kawakita et al., 2022).

## Discussion

The main outcome of this study is that multimodal neuroimaging, tractography-informed anisotropy and branched transport can be integrated into a single inverse framework that infers propagation architecture rather than presupposing it. The inferred object is not a trajectory, not a control signal and not a predefined connectome, but a *brain reaction map*: an effective routing backbone that connects stimulation to distributed response under anatomical and dynamical constraints.

Three conclusions emerge. First, multimodal estimation of source and target measures is both feasible and structurally informative. The combination of blood-oxygen-level-dependent spatial localisation and electrophysiological temporal resolution produces source and target measures that are sufficiently concentrated to drive a non-trivial transport problem while remaining robust to the fusion weight. Second, anisotropy matters at the architectural level. The anisotropic branched solution is not simply a weighted perturbation of the isotropic one; it activates a different routing logic, with stronger relay structure and clearer anatomical alignment. Third, dynamic plausibility does not reduce to geometric efficiency. The hybrid extension shows that the graphs preferred by transport cost alone need not minimise the dynamic control cost, and that rank reversals across $\lambda$ are intrinsic rather than exceptional.

The biological interpretability of the inferred architecture is an important point. In the present synthetic setting, the relay regions selected by the anisotropic BOT solution are not anonymous intermediates: the strongest bottlenecks and branching interfaces emerge preferentially in dorsal attention, salience/ventral attention, frontoparietal and thalamic nodes, that is, in systems commonly interpreted as integrative bridges between sensory input and distributed action or cognitive output (Bazinet et al., 2023; Fotiadis et al., 2024; Gilson et al., 2020; Patow et al., 2024). In this sense, the model does not simply recover short routes between sources and sinks; it selects a mesoscale backbone in which anatomically and functionally plausible association systems act as shared highways for signal aggregation before redistribution. Additional discussion of the mesoscale interpretation of relay systems is provided in Supplementary Note 5.

The interpretation of the Pareto frontier is also important for readers outside optimal transport or variational analysis. Each point in the $(E_\alpha ,J_{\textrm{dyn}})$ plane represents one candidate routing architecture, with one axis measuring geometric economy and the other dynamic steering cost. The frontier therefore marks the set of graphs that are optimal trade-offs between these two criteria. Rank reversals occur when the preferred architecture changes as the relative weight of dynamics is increased: a graph may be the most economical one geometrically, yet cease to be optimal once controllability is taken seriously. This is not a technical curiosity but a conceptual message: geometry and dynamics should be treated as coupled principles of large-scale propagation rather than as interchangeable surrogates.

Several limitations remain. The present article uses synthetic multimodal data and a low-dimensional cortical support with 18 regions of interest, the candidate graph is finite, and the stochastic dynamics is linear. The tractography prior is represented by a coarse tensor field rather than subject-specific whole-brain tract reconstruction. A further simplification concerns directionality in the graph-induced dynamics. Although the transport optimisation is posed on directed arcs and remains source–target oriented through the balance constraint $Aw=b$, the weighted adjacency matrix used in the stochastic extension is symmetrised before Laplacian construction. This choice should not be read as biological symmetry of the connectome. Rather, it reflects the fact that diffusion-MRI-based structural connectomes typically do not resolve axonal polarity in vivo, so the resulting structural prior is more naturally interpreted as an undirected coupling scaffold. Consequently, the present dynamic layer captures large-scale graph-mediated propagation on the inferred support, but it does not distinguish feedforward from feedback anatomical asymmetries. Extending the framework to genuinely directed structural priors is an important goal for future work. These simplifications are intentional: they isolate the effect of multimodal source–target inference, anisotropic geometry and hybrid selection in a transparent setting.

A related concern is scalability. The current implementation solves a constrained nonlinear optimisation problem with SLSQP on a sparse candidate graph and then estimates dynamic costs by Monte Carlo simulation. For the present proof-of-concept, this strategy is stable and sufficient. Scaling to human connectomes with 200 or more regions is conceptually straightforward but computationally more demanding, because the number of candidate arcs, optimisation variables and repeated dynamic evaluations all increase substantially. In practice, this points to several natural extensions: anatomically informed graph sparsification, continuation or warm-start strategies across $\alpha$, parallel evaluation of dynamic costs, and alternative large-scale optimisation schemes based on decomposition, proximal updates or differentiable surrogates. The current results should therefore be read not as a final engineering solution for whole-brain resolution, but as a variational proof-of-principle that motivates these larger-scale developments. Practical routes toward scaling the framework beyond the present 18-ROI proof-of-concept are discussed in Supplementary Note 6 and Supplementary Fig. S6.

More broadly, the framework suggests that a substantial part of what is often interpreted as state-transition difficulty may in fact be a question of architecture inference. If the propagation substrate itself is allowed to vary, then the correct inverse problem is no longer only to steer dynamics on a graph, but to determine which graph best explains how stimulation is transformed into reaction. In this sense, the main computational contribution of the present article should be read together with the theoretical foundation developed in the companion paper (Mendico, 2026).

## Methods

### Overview of the Computational Pipeline

The computational workflow consists of five successive stages: simulation of task-related blood-oxygen-level-dependent data, simulation of source-reconstructed electrophysiology, multimodal fusion of stimulation and reaction measures, construction of anisotropic transport costs from diffusion-informed tensors, and inference of a reaction map through branched transport optimisation followed by graph-induced stochastic analysis. All simulations were implemented in Python in a companion notebook.

### Synthetic Cortical Support and Region Layout

All computations were performed on a common support made of $N_{\textrm{roi}}=18$ regions of interest embedded in a two-dimensional cortical slice. Twelve regions were placed on an outer elliptical shell and six on an inner shell, producing a coarse geometry with peripheral sensory-association regions and inner relay regions. Regions were labelled to mimic visual, auditory, sensorimotor, dorsal attention, salience/ventral attention, limbic, frontoparietal, default mode and thalamic systems.

### Task-Based fMRI Simulation and General Linear Model

The fMRI acquisition was simulated over $T_{\textrm{fmri}}=300$ s with repetition time $TR=2.0$ s, yielding $N_{\textrm{vol}}=150$ volumes. Stimulus and reaction epochs followed a repeating block design consisting of 30 s stimulus, 30 s rest, 30 s reaction and 30 s rest. Neural task regressors were convolved with a canonical double-gamma haemodynamic response function

$$ h(t)= \frac{t^{a_1-1}e^{-t/b_1}}{\Gamma (a_1)b_1^{a_1}} - c\,\frac{t^{a_2-1}e^{-t/b_2}}{\Gamma (a_2)b_2^{a_2}}, \qquad t\ge 0, $$

with $a_1=6$, $a_2=16$, $b_1=b_2=1$, $c=1/6$, sampled on $[0,32]$ s and normalised to unit peak magnitude.

The design matrix was

$$ X_{\textrm{GLM}}= \bigl [x_{\textrm{stim}},x_{\textrm{react}},\textbf{1}\bigr ], $$

where $x_{\textrm{stim}}$ and $x_{\textrm{react}}$ denote the haemodynamically convolved task regressors and $\textbf{1}$ is the intercept. Region-wise blood-oxygen-level-dependent data were generated as

$$ Y=X_{\textrm{GLM}}\beta _{\textrm{true}}+\varepsilon , $$

with Gaussian noise of standard deviation $0.15$. Positive stimulus- and reaction-related contrast statistics were extracted as the fMRI activity scores.

### Source-Reconstructed EEG/MEG Simulation

Source-reconstructed electrophysiological data were simulated over a $T_{\textrm{eeg}}=1.0$ s epoch with sampling rate $f_s=512$ Hz, giving $N_{\textrm{samp}}=512$ samples per epoch. Sensor-level data were generated on $N_{\textrm{sensor}}=64$ sensors placed on a circle around the cortical support. The lead field $L\in \mathbb {R}^{N_{\textrm{sensor}}\times N_{\textrm{roi}}}$ was defined by inverse-square distance falloff between sensors and source regions and then normalised by its maximum entry.

Source reconstruction used a minimum-norm inverse

$$ L^\dagger = L^\top (LL^\top +\lambda _{\textrm{reg}}I)^{-1}, $$

with Tikhonov regularisation parameter $\lambda _{\textrm{reg}}=0.05$. Early stimulus-locked source components were placed in visual and auditory regions, whereas later reaction-locked components were placed in sensorimotor and default-mode-related regions. Forty noisy trials were generated and averaged to improve signal-to-noise ratio. Stimulation and reaction source scores were obtained from mean absolute source amplitudes over the windows [70, 200] ms and [300, 550] ms, respectively.

### Multimodal fusion of source and target measures

Let $a_i^{\textrm{fMRI,stim}}$, $a_i^{\textrm{fMRI,react}}$, $a_i^{\textrm{EEG,stim}}$, and $a_i^{\textrm{EEG,react}}$ denote the modality-specific regional scores after normalisation to unit maximum. The fused stimulation and reaction profiles were defined by weighted geometric averaging,

$$ a_i^{\textrm{stim}} = \bigl (a_i^{\textrm{fMRI,stim}}+\varepsilon _0\bigr )^{w_f} \bigl (a_i^{\textrm{EEG,stim}}+\varepsilon _0\bigr )^{1-w_f}, $$

$$ a_i^{\textrm{react}} = \bigl (a_i^{\textrm{fMRI,react}}+\varepsilon _0\bigr )^{w_f} \bigl (a_i^{\textrm{EEG,react}}+\varepsilon _0\bigr )^{1-w_f}, $$

with $w_f=0.55$ and $\varepsilon _0=10^{-6}$. The final source and target measures were

$$ \mu _{\textrm{stim}}^{+}(i)=\frac{a_i^{\textrm{stim}}}{\sum _j a_j^{\textrm{stim}}}, \qquad \mu _{\textrm{react}}^{-}(i)=\frac{a_i^{\textrm{react}}}{\sum _j a_j^{\textrm{react}}}, $$

and the supply–demand vector was $b=\mu _{\textrm{stim}}^{+}-\mu _{\textrm{react}}^{-}$.

Sensitivity of the fused stimulation measure to the fusion weight was assessed over a grid $w_f\in \{0,0.125,\dots ,1\}$.

### Diffusion-Informed Anisotropic Cost Construction

Synthetic diffusion tensors $D_i\in \mathbb {R}^{2\times 2}$ were assigned to each region of interest. Relay regions corresponding to dorsal attention, salience/ventral attention, frontoparietal and thalamic systems were given higher anisotropy, whereas other regions were assigned lower anisotropy. Fractional anisotropy was computed from the eigenvalues of each tensor.

A candidate graph was built by a $k$-nearest-neighbour rule with $k=5$, producing an undirected edge set that was duplicated into directed arcs. For an arc $e=(i,j)$ with tangent direction $\tau _{ij}$ and Euclidean length $\ell _{ij}$, the anisotropic edge cost was defined by midpoint evaluation:

$$ \beta _{ij}^{\textrm{aniso}} = \ell _{ij} \sqrt{ \tau _{ij}^{\top } \bigl (D_{ij}^{\textrm{mid}}+\varepsilon I\bigr )^{-1} \tau _{ij} }, $$

where $D_{ij}^{\textrm{mid}}=\frac{1}{2}(D_i+D_j)$ and $\varepsilon =0.05$. For comparison, an isotropic baseline cost was also constructed from Euclidean distance and a scalar white-matter score.

### Discrete branched transport optimisation

Let $A$ denote the incidence matrix of the directed candidate graph. For a fixed branching exponent $\alpha \in (0,1)$, the optimisation problem was

$$ \min _{w\ge 0}\; \sum _{e\in \mathcal {E}}\beta _e\,w_e^\alpha \qquad \text {subject to}\qquad Aw=b. $$

The principal analyses used $\alpha =0.65$. Optimisation was performed with SLSQP and 12 random restarts. Each restart was initialised from a combination of a clipped least-squares solution of the balance constraints and a small positive random perturbation. After optimisation, fluxes below $10^{-4}$ times the maximal flux were set to zero for support visualisation only. The same candidate graph and optimisation procedure were used for both isotropic and anisotropic costs.

### Graph-Induced Stochastic Dynamics and Dynamic Cost

The anisotropic optimal graph was converted into a directed weighted adjacency matrix $W$, then symmetrised to

$$ S=\frac{1}{2}(W+W^\top ). $$

This symmetrisation is used only for the Laplacian-based stochastic extension. It should not be interpreted as a claim that macroscale structural brain connectivity is biologically symmetric; rather, it reflects the fact that diffusion-MRI tractography does not directly resolve axonal polarity in vivo, so undirected or symmetrised structural couplings remain a standard approximation at this level of description. The weighted degree vector $d$ and graph Laplacian $L$ were defined by

$$ d_i=\sum _j S_{ij}, \qquad L=\textrm{diag}(d)-S. $$

The graph-induced stochastic dynamics was

$$ dX_t = A_{G,w}X_t\,dt + B_{\textrm{stim}}a_t\,dt + u_t\,dt + C_{G,w}\,dW_t, $$

with

$$ A_{G,w}=-\kappa I-\beta _{\textrm{dyn}}L, \qquad C_{G,w}=\sigma _0 I+\sigma _1 \textrm{diag}(\sqrt{d}). $$

The parameters were $\kappa =0.8$, $\beta _{\textrm{dyn}}=0.4$, $\sigma _0=0.08$, and $\sigma _1=0.04$. The simulation horizon was $T_{\textrm{sim}}=3.0$ s with timestep $dt=0.005$, giving $N_{\textrm{steps}}=600$ steps. Path ensembles contained $N_{\textrm{paths}}=80$ trajectories.

The uncontrolled process corresponded to $u_t\equiv 0$. The controlled process used a bridge-type feedback toward the target profile,

$$ u_t = C_{G,w}C_{G,w}^{\top } \frac{m_T-X_t}{T_{\textrm{sim}}-t+\varepsilon _{\textrm{bridge}}}, $$

with $\varepsilon _{\textrm{bridge}}=0.05$. Dynamic cost was estimated by Monte Carlo approximation of the quadratic control functional associated with the controlled process.

### Pareto and $\lambda$-analysis

The anisotropic transport problem was repeated over a grid of branching exponents

$$ \alpha \in \{0.20,0.20+\Delta ,\dots ,0.92\}, $$

with 22 evenly spaced values. For each inferred graph, the dynamic cost $J_{\text {dyn}}$ was estimated numerically. The hybrid functional

$$ F_\lambda (G,w)=E_\alpha (G,w)+\lambda J_{\textrm{dyn}}(G,w) $$

was evaluated over $\lambda \in [0,6]$ on a grid of 300 values. Pareto points were obtained from the set of $(E_\alpha ,J_{\textrm{dyn}})$ pairs and rank reversals were identified by sign changes in pairwise differences of the corresponding $F_\lambda$ curves.

The branching exponent $\alpha \in (0,1)$ controls the degree of aggregation in the inferred transport architecture and is therefore treated as a model-selection parameter rather than a fixed constant. In practical applications, $\alpha$ can be selected based on two complementary criteria. First, a stability-based approach consists in identifying a range of $\alpha$ values for which the inferred reaction map remains structurally robust under small perturbations of $\alpha$, as assessed for example by the behaviour of global observables and supports across the $\alpha$-grid (see Supplementary Figs. 7–8). Second, when the graph-induced stochastic extension is considered, $\alpha$ can be chosen by evaluating the geometric–dynamical trade-off, either through the Pareto frontier in the $(E_\alpha , J_{\textrm{dyn}})$ plane or by minimizing the hybrid functional $F_\lambda$ for a given application-dependent value of $\lambda$. This viewpoint treats $\alpha$ as indexing a family of candidate routing architectures, from which one selects a stable and dynamically plausible solution rather than fixing a priori a single value.

## Supplementary Information

Below is the link to the electronic supplementary material.

Supplementary file 1 (pdf 1314 KB)

## Data Availability

This study uses synthetic multimodal datasets gener- ated within the companion computational notebook. All scripts required to reproduce the simulations, figures and derived measures will be made publicly available upon publication.

## Code Availability

The full pipeline, including fMRI simulation, source-reconstructed EEG/MEG, multimodal fusion, anisotropic branched transport optimisation and stochastic graph dynamics, will be deposited in a public repository upon publication.
