# Supplementary material for: Multimodal Branched Transport Infers Anatomically Aligned Brain Reaction Maps
Source: Neuroinformatics. 2026 Jun 10;24(2):34. doi: 10.1007/s12021-026-09791-4 (PMC13253786; doi:10.1007/s12021-026-09791-4)
Supplement: Supplementary file 1 — (pdf 1314 KB) [file 12021_2026_9791_MOESM1_ESM.pdf]

Supplementary overview

This Supplementary Information is designed to reinforce the central claims of the main manuscript along four dimensions that are especially relevant for evaluation by a broad multidisciplinary readership. First, it examines how the inferred anisotropic routing architecture varies with the branching exponent α, thereby clarifying that the reported reaction maps do not arise from a single isolated parameter choice. Second, it compares the anisotropic branched solution with simpler non-branched baselines, making explicit which features of the inferred architecture are genuinely due to the ramified objective. Third, it quantifies the relay-region structure of the inferred map, thereby strengthening the biological interpretability of the selected transport backbone. Fourth, it provides a proof-of-principle scalability analysis, addressing the natural question of whether the present framework can plausibly extend beyond the 18-ROI synthetic demonstration.

Taken together, these supplementary analyses support four stronger versions of the claims made in the main text. The first is that the anisotropic reaction map is *robust across a stable branching regime*, rather than being a fragile solution at one preferred α. The second is that the emergence of *shared relay corridors* is a distinctive signature of branched transport and is not reproduced by simpler linear-cost or shortest-path surrogates. The third is that the strongest relay regions are *biologically interpretable mesoscale bottlenecks*, not anonymous graph intermediates. The fourth is that the current implementation should be read as a *variational proof-of-principle with plausible computational paths to scale-up*, rather than as an already final large-connectome engineering solution.

Supplementary Note 1. Extended dependence on the branching exponent α

A defining ingredient of the model is the concavity of the edge cost w_e^α, with $0 < \alpha < 1$. Smaller values of α favour stronger route sharing and earlier aggregation of flux; values closer to 1 weaken the ramification incentive and produce more distributed routing. In the main manuscript, the principal reaction maps are shown for a representative value $\alpha = 0.65$. Here we make explicit how the global observables and inferred supports behave across the broader explored regime.

Global observables across the α-grid

Supplementary Fig. 1 reports the anisotropic geometric cost E_α, the dynamic cost J_{dyn}, and the support size $|e^*|$ over the full α-grid. The dominant trend away from the transition region is clear: the geometric cost decreases as α increases, while the support size remains in a relatively stable range. The dynamic cost varies much more weakly over the stable regime than the geometric cost, which is one of the reasons why the Pareto structure in the main manuscript is non-trivial.

A particularly important feature is the sharp anomaly around $\alpha \approx 0.4$, where both the geometric and dynamic observables undergo abrupt changes and the support collapses. We interpret this interval as a *near-degenerate transition regime* of the optimisation landscape rather than as the biologically relevant operating point of the framework. This regime is scientifically informative: it indicates that the model separates naturally into weakly branched and strongly branched phases, with a narrow transition region between them. However, the main conclusions of the article do not rely on that transition itself, but on the stable family of solutions around and above the main-text reference value $\alpha = 0.65$.

Representative anisotropic maps across branching regimes

Supplementary Fig.2 shows anisotropic reaction maps for four representative values of α: 0.25, 0.45, 0.65, and 0.85. The figure makes the branching logic visually transparent. Lower α values produce stronger aggregation and a smaller number of dominant high-load corridors, whereas larger α values support more distributed routing across the graph. At the same time, the large-scale organisation of the backbone remains recognisable across the stable range, indicating that the main pathways are not completely rearranged by modest changes in branching strength.

This is important for interpretation. The branching exponent does not merely rescale flux magnitudes; it changes the architecture itself. The reaction map should therefore be understood as belonging to a parameterised family of economical routing backbones, within which the main-text solution at $\alpha = 0.65$ occupies an intermediate and structurally stable regime.

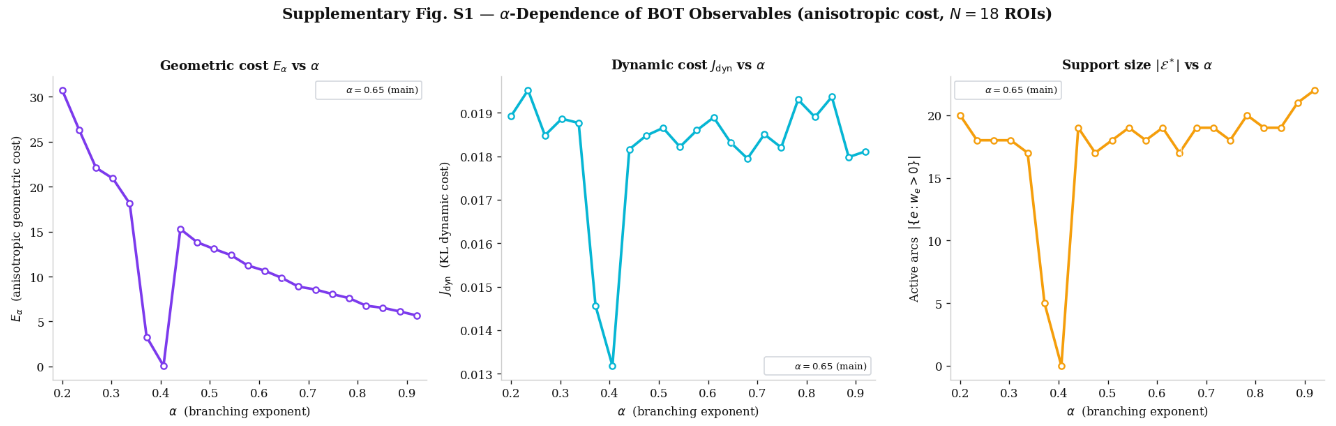

Figure 1: α-dependence of anisotropic branched optimal transport observables. The anisotropic branched transport problem was solved over a grid of branching exponents α in order to assess the robustness of the inferred reaction maps. The left panel shows the anisotropic geometric transport cost E_α as a function of α. The middle panel shows the corresponding dynamic cost J_{dyn} estimated from the graph-induced stochastic extension. The right panel shows the number of active arcs in the inferred support. The vertical dashed line marks the main-text reference value $\alpha = 0.65$. Away from a narrow near-degenerate transition region around $\alpha \approx 0.4$, the observables vary in a structured manner and the support size remains within a stable range. This analysis indicates that the main reaction map is not an isolated parameter-specific solution, but belongs to a stable family of anisotropic branched routing architectures.

Table 1: **Supplementary Table S1. Representative anisotropic supports across branching regimes.**

α	Active arcs	Qualitative regime
0.25	17	strong aggregation
0.45	19	post-transition stable regime
0.65	18	main-text reference regime
0.85	20	more distributed routing

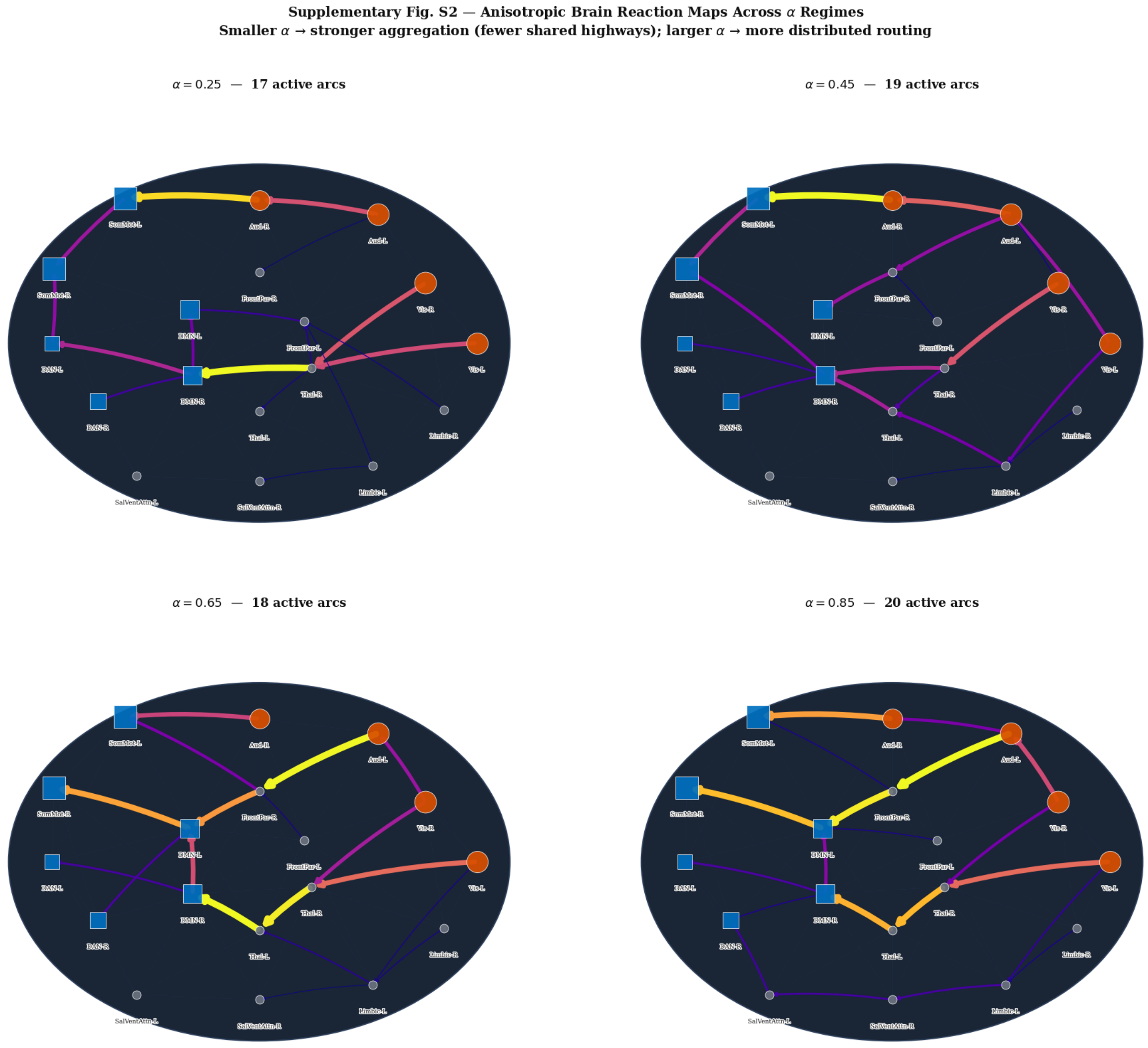

Figure 2: Anisotropic brain reaction maps across branching regimes. Representative anisotropic branched transport solutions are shown for four values of the branching exponent, $\alpha = 0.25$, $\alpha = 0.45$, $\alpha = 0.65$, and $\alpha = 0.85$. Smaller values of α impose a stronger concavity in the transport cost and therefore favour earlier aggregation of flux into a smaller number of shared high-load corridors. Larger values of α weaken the branching incentive and produce more distributed routing over the candidate graph. The solution at $\alpha = 0.65$ corresponds to the main-text reference regime. Although the degree of aggregation changes with α, the dominant anisotropic backbone remains interpretable across the stable parameter range, supporting the view that the inferred brain reaction map should be understood as a member of a structured family of routing architectures.

Supplementary Note 2. Comparison with non-branched baselines

A critical question for the interpretation of the main results is whether the inferred shared corridors are a distinctive outcome of anisotropic branched transport or whether they would also emerge under simpler routing principles. Supplementary Fig. 3 addresses this by comparing the anisotropic BOT solution with two non-branched surrogates defined on the same source–target pair and the same candidate graph: an anisotropic linear-cost transport surrogate ($\alpha = 1$) and a greedy shortest-path surrogate.

What the baseline comparison shows

The comparison reveals a clear structural hierarchy. The anisotropic BOT solution concentrates transport onto a relatively small set of shared relay corridors. The linear-cost surrogate weakens this concentration and spreads transport over a broader active support. The shortest-path surrogate fragments routing even further, favouring direct pairwise routes over mesoscale redistribution. Thus, the *shared-highway architecture* that motivates the main manuscript is not simply a graph sparsity effect, nor a generic consequence of anisotropic weights. It is the specific consequence of combining anisotropy with a concave transport objective.

This distinction matters because it clarifies the conceptual gain over standard transport or routing baselines. The

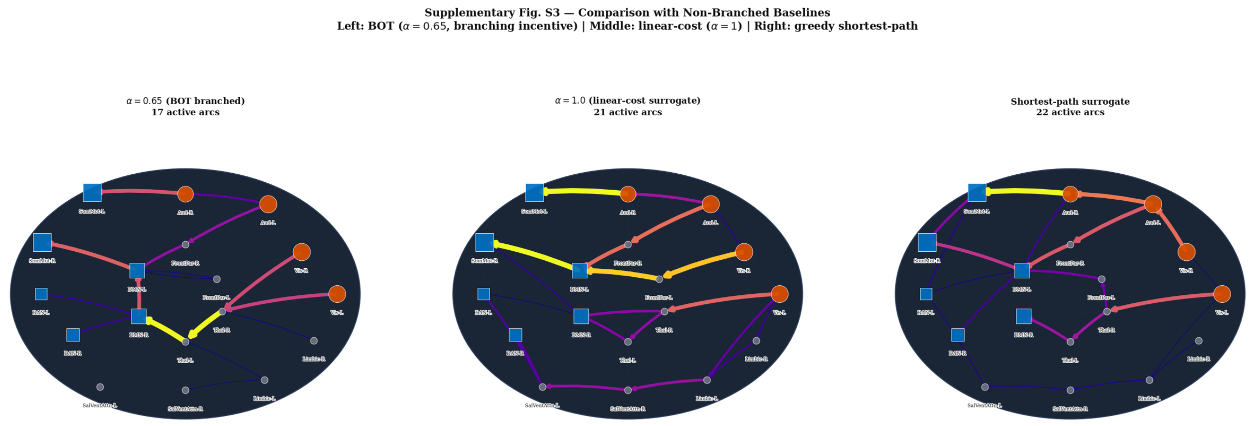

Figure 3: Comparison with non-branched routing baselines. The anisotropic branched optimal transport solution is compared with two simpler non-branched surrogates defined on the same source–target measures and candidate graph. The left panel shows the anisotropic branched solution with $\alpha = 0.65$, which concentrates transport onto shared relay corridors. The middle panel shows an anisotropic linear-cost transport surrogate corresponding to $\alpha = 1$, for which the concavity-driven incentive to merge flux is absent. The right panel shows a greedy shortest-path surrogate, which favours direct pairwise routing and produces a more fragmented transport pattern. The comparison demonstrates that the shared-highway structure observed in the main model is not merely a consequence of graph sparsity or anisotropic edge weights. Rather, it arises from the combination of anatomical anisotropy with the concave branched transport objective.

branched formulation does not only connect sources to sinks cheaply; it explains why separate signal contributions may become temporarily co-routed through common mesoscale corridors before diverging toward reaction-producing regions.

Supplementary Note 3. Relay-region statistics and mesoscale interpretation

The main manuscript argues that the anisotropic solution is biologically interpretable because the strongest relay regions are not arbitrary graph intermediates. Supplementary Fig. 4 and Supplementary Table 2 make this point quantitative.

Dominant relay systems

The left panel of Supplementary Fig. 4 ranks the top relay nodes by relay score. The strongest relays are the bilateral thalamic nodes, followed by bilateral default-mode and frontoparietal nodes. The right panel shows node-level incoming and outgoing flux, distinguishing relay, source, and sink nodes. Relay nodes lie near the diagonal because they both receive and redistribute substantial flow, whereas source- and sink-dominated nodes are displaced toward input- or output-heavy regimes.

This structure is highly informative. A direct-routing model might have concentrated almost all load on dominant source and target regions themselves. Instead, the branched anisotropic solution identifies intermediate systems that act as economical redistribution bottlenecks. In the present synthetic setting, the bilateral thalamic, default-mode, and frontoparietal nodes therefore emerge as the principal mesoscale bridge systems of the inferred reaction map.

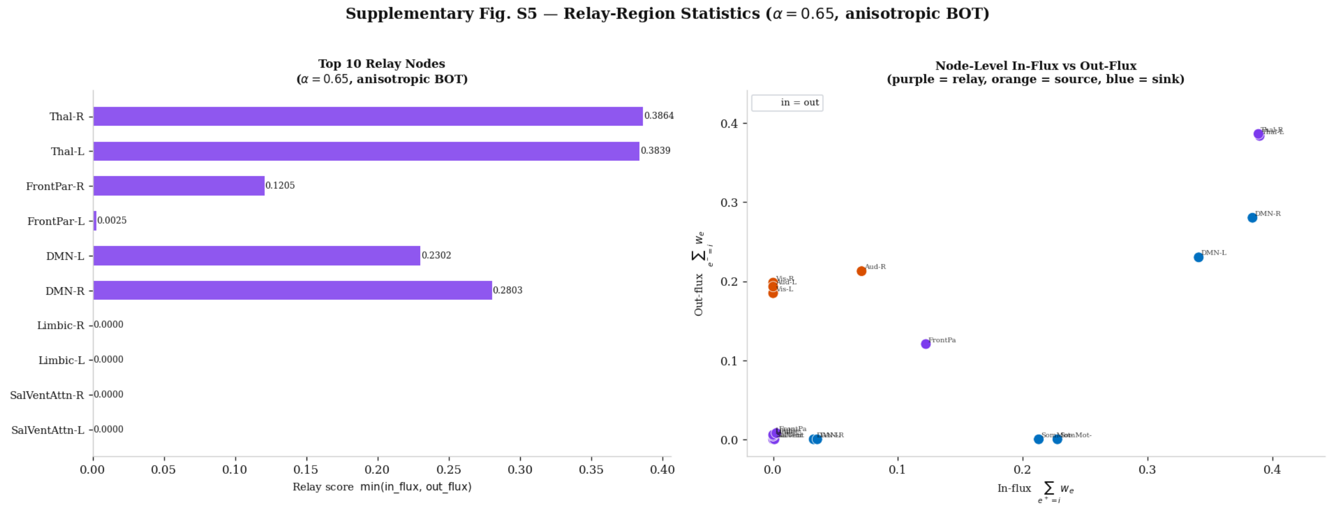

Figure 4: Relay-region statistics for the anisotropic solution at $\alpha = 0.65$. Relay structure was quantified for the main anisotropic branched transport solution. The left panel ranks regions by relay score, identifying nodes that carry substantial intermediate flux and therefore act as candidate aggregation or redistribution sites. The strongest relay scores are observed in bilateral thalamic regions, followed by default-mode and frontoparietal nodes. The right panel shows node-level incoming and outgoing fluxes, with relay nodes distinguished from source-dominated and sink-dominated regions. Relay nodes lie near the diagonal because they both receive and redistribute substantial flow, whereas source and sink nodes are biased toward outgoing or incoming flux, respectively. These results show that the inferred transport backbone is not simply a collection of direct source-to-target routes, but contains biologically interpretable mesoscale bottlenecks that mediate signal aggregation before redistribution.

Table 2: Supplementary Table S2. Dominant relay nodes for the anisotropic solution at $\alpha = 0.65$.

Node	Relay score
Thal-R	0.3864
Thal-L	0.3839
DMN-R	0.2803
DMN-L	0.2302
FrontPar-R	0.1205
FrontPar-L	0.0025
Limbic-L	0.0000
Limbic-R	0.0000
SalVentAttn-R	0.0000
SalVentAttn-L	0.0000

The dominance of bilateral thalamic and default-mode nodes is especially relevant because it indicates that the inferred architecture is not merely a shortest transfer from sensory entry to motor output. Instead, the model selects a hierarchical backbone in which integrative bridge systems mediate aggregation and redistribution at a mesoscale level.

Supplementary Note 4. Scalability of the anisotropic branched optimal transport framework

A natural concern for the present proof-of-concept study is whether the anisotropic branched optimal transport framework can be extended beyond the small regional support used in the main analysis. The main manuscript deliberately uses an 18-region cortical support in order to keep the complete multimodal pipeline transparent: source–target estimation, anisotropic cost construction, nonlinear branched transport optimisation, and graph-induced stochastic analysis can all be inspected directly at this scale. However, the variational formulation is not intrinsically tied to this low-dimensional example. What matters computationally is the size and sparsity of the candidate graph, the number of optimisation variables, and the stability of the inferred active support as the number of nodes increases. To address this point, we performed an additional scalability experiment on synthetic sparse graphs of increasing size.

The aim of this analysis is not to establish a universal asymptotic complexity result. The current implementation uses a general constrained nonlinear optimiser and is therefore not intended as a final large-connectome production

solver. Rather, the purpose is to test whether the qualitative signatures of the proposed framework persist when the spatial resolution is increased while preserving a sparse candidate-graph structure. In particular, we ask whether the solver remains practically usable over a larger range of synthetic graph sizes, whether the candidate graph remains sparse under a fixed-degree construction, whether the optimal BOT support remains a minority subset of all available arcs, and whether relay-mediated organisation persists beyond the 18-node demonstration.

For each graph size, we generated a synthetic cortical layout and constructed a sparse k-nearest-neighbour graph with $k = 5$. The undirected neighbourhood graph was then duplicated into directed arcs, as in the main manuscript, so that the transport problem could be formulated with an oriented incidence matrix. Source and target nodes were assigned according to the same conceptual source–reaction interpretation used throughout the paper: source nodes represent stimulation-related mass, target nodes represent reaction-related mass, and the optimisation selects a routing architecture connecting the two under anisotropic geometric costs. For each value of N, we solved the anisotropic BOT problem

$$\min_{w \geq 0} \sum_{e \in E} \beta_e w_e^{\alpha} \qquad \text{subject to} \qquad Aw = b,$$

with branching exponent $\alpha = 0.65$. The optimisation was repeated with multiple random restarts for each graph size, and the reported quantities summarise the resulting solver behaviour and graph structure.

Supplementary Fig. 5 summarises the results. Panel A reports the wall-clock runtime of the BOT solver as a function of the number of nodes on a log–log scale. Over the tested range, the empirical fit indicates a super-quadratic scaling trend, approximately

$$t_{\text{solve}} \propto N^{2.58}.$$

This confirms that the current implementation becomes more expensive as the graph size increases, as expected for a constrained nonlinear optimisation problem. At the same time, the observed growth remains controlled over the explored synthetic scales. This scaling should not be interpreted as a theoretical complexity bound: it depends on the present solver, graph family, initialisation strategy, stopping criteria, and restart protocol. Its role is instead to provide an empirical indication that the framework can be executed on substantially larger sparse graphs than the 18-region example used for the main multimodal demonstration.

Panel B shows the number of directed arcs in the candidate graph as a function of N. Because the graph is built using a fixed-degree k-nearest-neighbour rule, the number of candidate arcs grows approximately linearly,

$$|E| \propto N^{1.00}.$$

This point is important for interpretation. The increase in computational cost is not caused by a transition toward dense all-to-all connectivity; it reflects the increasing number of variables and constraints in a sparse nonlinear transport problem. Thus, graph sparsity is preserved by construction as the resolution increases. This is the natural regime for connectomic applications, where anatomical or tractography-informed priors can be used to restrict candidate propagation paths before solving the transport problem.

Panel C reports the fraction of active arcs in the optimal BOT support. Across the tested graph sizes, only a small fraction of candidate arcs carries non-negligible flux, with an average active fraction close to 0.15. Thus, increasing the graph resolution does not cause the inferred reaction map to spread diffusely over the full candidate graph. Instead, the concave transport objective continues to select a reduced set of preferred propagation corridors. This behaviour is consistent with the central mechanism of branched transport: because $w \mapsto w^{\alpha}$ is concave for $0 < \alpha < 1$, the optimisation favours aggregation of flow along shared routes rather than independent routing through many separate paths.

Panel D shows the normalised transport cost E_{α}/N. The decreasing trend indicates that larger graphs provide additional routing flexibility per node. As the number of admissible intermediate points increases, the solver can exploit more efficient aggregation and redistribution patterns. In this sense, increasing the graph resolution does not merely make the optimisation problem larger; it also enriches the class of admissible reaction-map architectures. The decrease of E_{α}/N should therefore be interpreted geometrically, as evidence that finer sparse supports allow the branched transport mechanism to identify more economical paths through intermediate relay structures.

Panels E and F quantify the relay organisation of the inferred solutions. Panel E reports the mean relay score among the strongest relay nodes, while Panel F reports the Gini coefficient of the relay-score distribution. The persistence of non-zero top-relay scores across graph sizes indicates that relay-like bottlenecks are not an artefact of the smallest synthetic example. At the same time, the relay-score Gini coefficient remains at intermediate-to-high values, showing that transport load is heterogeneously distributed rather than uniformly spread over all nodes. The inferred architectures therefore continue to display mesoscale organisation: a subset of nodes acts as aggregation or redistribution hubs, while the remaining nodes carry weaker or more peripheral flow.

Panels G–I display representative reaction maps for $N = 18$, $N = 72$, and $N = 288$. At $N = 18$, the solution reproduces the sparse regime used in the main text. At $N = 72$, the support becomes richer while preserving a clear branched structure. At $N = 288$, the graph contains many more possible locations and candidate arcs, but the optimal support still occupies only a minority subset of the available directed edges. In the displayed examples, the

number of active arcs increases from 9/114 for $N = 18$, to 72/410 for $N = 72$, and to 269/1536 for $N = 288$. Thus, the support becomes more detailed at higher resolution without becoming dense or visually uninterpretable. This is precisely the behaviour expected from a scalable branched-transport architecture: increasing resolution refines the inferred propagation backbone, but the concavity of the cost preserves sparse corridor selection.

Taken together, these results support three conclusions. First, the anisotropic BOT framework is not intrinsically restricted to the 18-node proof-of-concept used in the main manuscript. The present implementation can be run on substantially larger synthetic sparse graphs, and the inferred supports remain sparse over the tested range. Second, the main qualitative signatures of the method persist across scales: sparse active supports, branched routing corridors, relay-mediated organisation, heterogeneous relay load, and decreasing normalised transport cost. Third, the current solver should be understood as a transparent variational proof-of-principle rather than as a final engineering solution for high-resolution whole-brain connectomes.

Several natural computational extensions follow from this analysis. For subject-specific or cohort-level connectomic applications, candidate arcs should be restricted using anatomically informed sparsification, for example by combining spatial proximity with tractography-derived structural priors. Continuation or warm-start strategies across neighbouring values of α could reduce the cost of parameter exploration. Dynamic-cost evaluations for different inferred graphs can be parallelised, since they are independent once the transport supports have been computed. Finally, large-scale optimisation methods tailored to sparse nonlinear transport problems—including decomposition methods, proximal schemes, differentiable surrogates, or specialised nonconvex network-flow solvers—could substantially improve performance beyond the present SLSQP-based proof-of-concept. The scalability experiment therefore does not close the computational question; rather, it identifies a plausible path from the present variational demonstration toward larger connectome-level implementations.

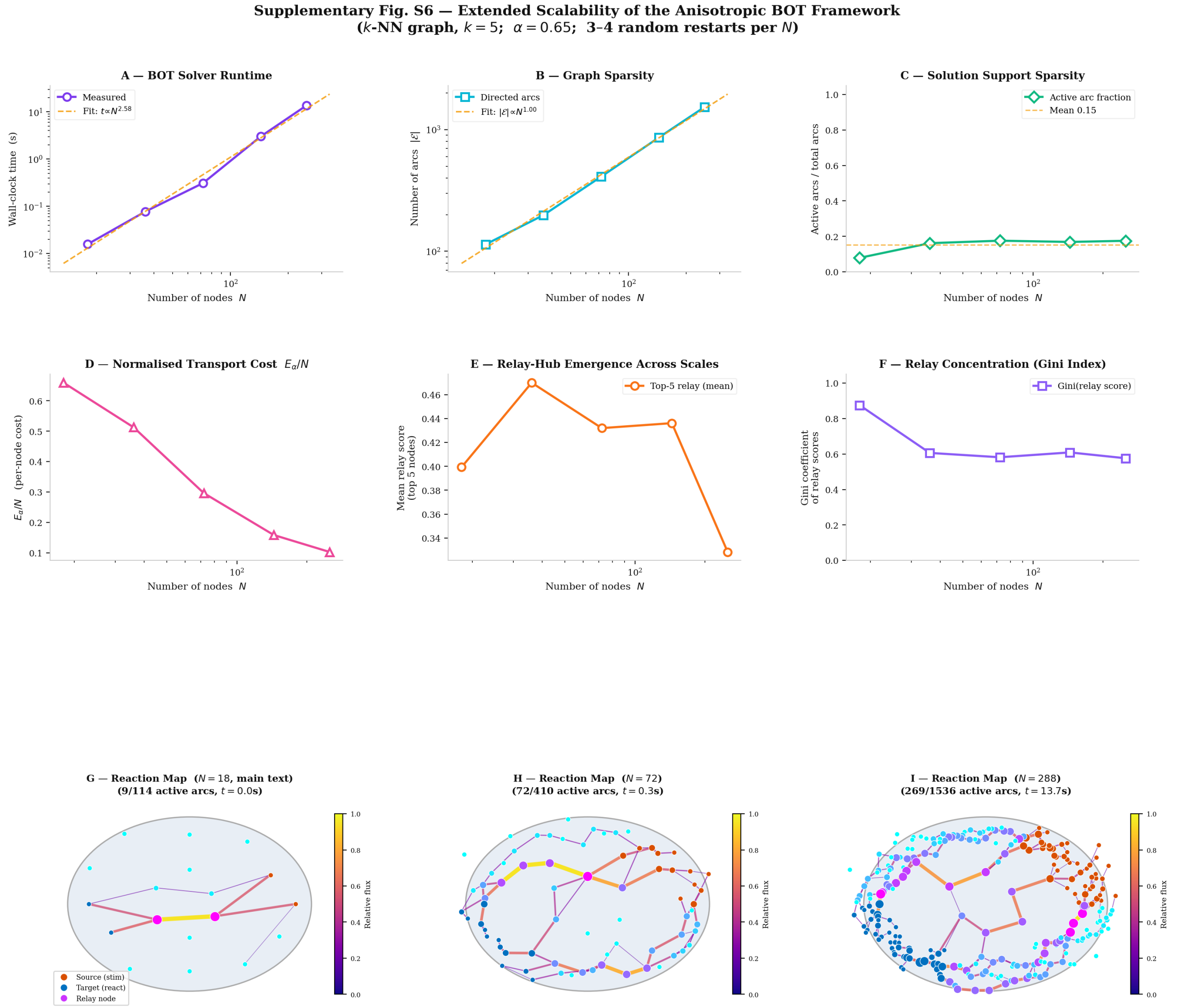

Figure 5: Extended scalability of the anisotropic branched optimal transport framework. The anisotropic BOT solver was evaluated on synthetic sparse graphs of increasing size in order to assess whether the framework remains computationally and structurally meaningful beyond the small proof-of-concept example considered in the main manuscript. All graphs were constructed using a k-nearest-neighbour rule with $k = 5$, the branching exponent was fixed to $\alpha = 0.65$, and each optimisation was performed with multiple random restarts. **(A)** Wall-clock runtime of the BOT solver as a function of the number of nodes N on a log–log scale. The empirical fit indicates a super-quadratic scaling trend, approximately $t_{\text{solve}} \propto N^{2.58}$ over the tested range. This trend should be interpreted as an empirical observation for the present implementation and graph family, rather than as a universal complexity bound. **(B)** Number of directed arcs in the candidate graph as a function of N. The fitted trend is approximately linear, $|E| \propto N^{1.00}$, confirming that the k-nearest-neighbour construction preserves graph sparsity as the number of nodes increases. **(C)** Fraction of active arcs in the optimal BOT support. Across scales, only a small fraction of candidate arcs carries non-negligible flux, with an average active fraction close to 0.15. Thus, increasing graph resolution does not produce dense or uninterpretable transport supports. **(D)** Normalised transport cost E_α/N. The decreasing trend indicates that larger graphs provide additional routing flexibility, allowing the solver to identify more efficient aggregation and redistribution pathways per node. **(E)** Mean relay score of the strongest relay nodes. High-scoring relay nodes persist across graph sizes, showing that the emergence of relay-like bottlenecks is not restricted to the smallest synthetic example. **(F)** Gini coefficient of the relay-score distribution. The coefficient remains at intermediate-to-high values, indicating that relay load is heterogeneous rather than uniformly distributed across all nodes. **(G–I)** Representative reaction maps for $N = 18$, $N = 72$, and $N = 288$, respectively. Source nodes, target nodes, relay nodes, and transport edges are shown together with a colour scale indicating relative flux. The inferred supports remain sparse while becoming progressively richer as the spatial resolution increases. In the displayed examples, the number of active arcs grows from $9/114$ for $N = 18$, to $72/410$ for $N = 72$, and to $269/1536$ for $N = 288$. Together, these panels show that the anisotropic BOT framework preserves its main qualitative features across scales: sparse support, branched routing, relay-mediated organisation, heterogeneous relay concentration, and improved routing flexibility at higher resolution.

Supplementary Note 5. Strengthened interpretation of the supplementary analyses

Taken together, the supplementary analyses reinforce the main manuscript in four decisive ways.

First, the α-sensitivity analysis shows that the anisotropic reaction map belongs to a structured family of routing architectures, rather than depending on one isolated parameter choice. Second, the baseline comparison makes explicit that the shared relay corridors of the main manuscript are a genuine signature of branched transport and are not recovered by standard non-branched alternatives. Third, the relay statistics demonstrate that the strongest intermediates are biologically interpretable mesoscale systems rather than anonymous connectors. Fourth, the scalability analysis clarifies that the present work is best understood as a variational proof-of-principle that opens the way to larger connectomic implementations.

The purpose of this Supplementary Information is therefore not merely additive. It sharpens the paper scientific position as a proof-of-concepts of the theoretical paper [14]. The main result is not just that one can solve a synthetic transport problem on a small graph, but that a multimodal anisotropic branched-transport framework can reveal interpretable routing backbones, distinguish them from simpler baselines, and organise them into a geometric–dynamic trade-off landscape whose structure persists across a stable branching regime.
